# The Sigma-1 receptor is an ER-localized type II membrane protein

**DOI:** 10.1016/j.jbc.2021.101299

**Published:** 2021-10-11

**Authors:** Neeraj Sharma, Chaitanya Patel, Marina Shenkman, Amit Kessel, Nir Ben-Tal, Gerardo Z. Lederkremer

**Affiliations:** 1 The Shmunis School of Biomedicine and Cancer Research, Cell Biology Division, George Wise Faculty of Life Sciences, Tel Aviv University, Tel Aviv, Israel; 2 Sagol School of Neuroscience, Tel Aviv University, Tel Aviv, Israel; 3 School of Neurobiology, Biochemistry and Biophysics, George Wise Faculty of Life Sciences, Tel Aviv University, Tel Aviv, Israel

**Keywords:** sigma-1 receptor, endoplasmic reticulum, ER stress, neurodegeneration, membrane topology, cell surface, BAP, biotin acceptor peptide, CNX, calnexin, ER, endoplasmic reticulum, MAM, mitochondrion-associated membranes, S1R, Sigma-1 receptor, Strep, streptavidin, sulfo-NHS-LC-Biotin, sulfosuccinimidyl-6-(biotinamido)hexanoate, TG, thapsigargin, TMD, transmembrane domain

## Abstract

The Sigma-1 receptor (S1R) is a transmembrane protein with important roles in cellular homeostasis in normal physiology and in disease. Especially in neurodegenerative diseases, S1R activation has been shown to provide neuroprotection by modulating calcium signaling, mitochondrial function and reducing endoplasmic reticulum (ER) stress. S1R missense mutations are one of the causes of the neurodegenerative Amyotrophic Lateral Sclerosis and distal hereditary motor neuronopathies. Although the S1R has been studied intensively, basic aspects remain controversial, such as S1R topology and whether it reaches the plasma membrane. To address these questions, we have undertaken several approaches. C-terminal tagging with a small biotin-acceptor peptide and BirA biotinylation in cells suggested a type II membrane orientation (cytosolic N-terminus). However, N-terminal tagging gave an equal probability for both possible orientations. This might explain conflicting reports in the literature, as tags may affect the protein topology. Therefore, we studied untagged S1R using a protease protection assay and a glycosylation mapping approach, introducing N-glycosylation sites. Both methods provided unambiguous results showing that the S1R is a type II membrane protein with a short cytosolic N-terminal tail. Assessments of glycan processing, surface fluorescence-activated cell sorting, and cell surface biotinylation indicated ER retention, with insignificant exit to the plasma membrane, in the absence or presence of S1R agonists or of ER stress. These findings may have important implications for S1R-based therapeutic approaches.

The Sigma-1 receptor (S1R) is a small transmembrane protein (25 KDa), which is localized predominantly in the endoplasmic reticulum (ER), preferentially at the mitochondrion-associated membranes (MAM) (1, 2, 3, 4, 5). The S1R is expressed in many tissues and has important roles in modulating calcium signaling and mitochondrial function, with special importance in the central nervous system. Decreased levels of S1R or of its activity have been found associated with neurodegenerative diseases (6, 7, 8, 9, 10, 11), and S1R activation by agonists is neuroprotective in several of these diseases (12, 13, 14, 15). However, the basic topology of this protein remains controversial. Initial studies suggested the existence of two transmembrane domains (TMDs) (4). The determination of the crystal structure of the S1R points to only one TMD near the N-terminus of the protein, but still leaves open the possibility of two possible orientations in the membrane. There is no cleaved signal peptide, but the C-terminal bulk of the protein could be in the lumen of the ER (type II membrane protein) or in the cytosol (type III membrane protein).

Another important issue is to what extent the S1R reaches the cell surface. The S1R appears to be mainly localized at an ER subdomain that contacts mitochondria, the MAM (4). However, it has also been reported to be present on the plasma membrane (16) where it might be translocated upon activation with agonists (17). However, on closer inspection, the location seems to be not at the plasma membrane but at close subsurface areas (17, 18), perhaps at ER domains adjacent to the plasma membrane (19).

Given the biological and pharmacological importance of the S1R, a clear understanding of its topology relative to the membrane and of its presence or absence at the cell surface is essential. Prediction algorithms of S1R membrane topology give a wide array of possibilities, whereas the conflicting studies use tags that might affect S1R topology. Here we analyzed S1R topology using different approaches, some of them with untagged S1R. In one such approach, we introduced N-glycosylation sites at key residues and tested their glycosylation. These constructs also allowed us to follow their sugar chain processing as an indicator of ER exit.

## Results

### *In vivo* biotinylation of BAP-tagged S1R

**Figure 1:**
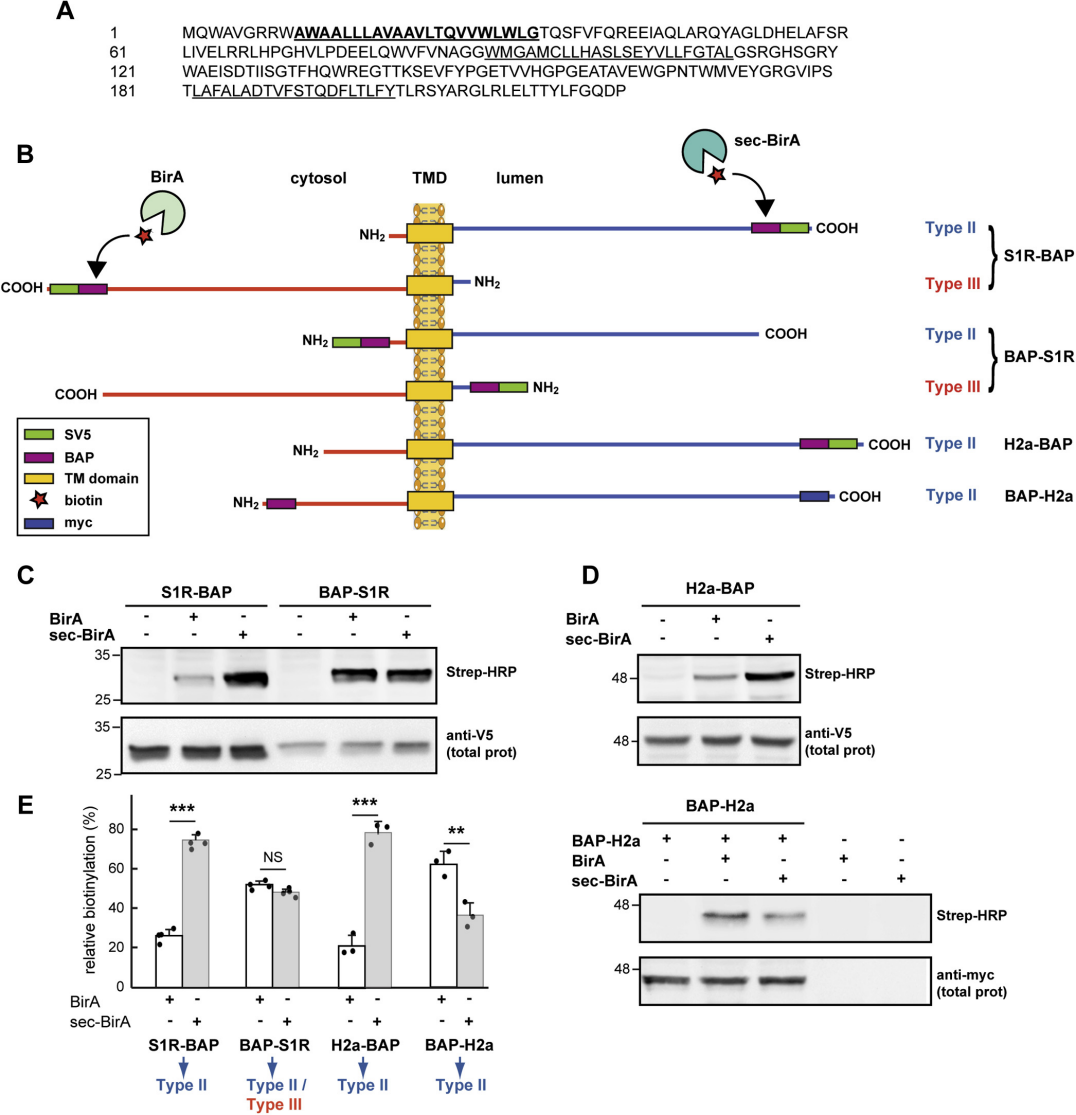
**C-terminal BAP-tagging of the S1R suggests a type II membrane topology and mixed topology by N-terminal tagging.***A*, sequence of human S1R. *Underlined* are hydrophobic stretches, in *bold* a region with a high probability prediction for a TMD and not bold for additional lower probability TMDs. *B*, a scheme of BAP-tagged S1R constructs S1R-BAP and BAP-S1R adopting type II or type III orientations, and their biotinylation in each case by cytosolic BirA or luminal sec-BirA. For comparison, BAP-tagged constructs of H2a, which has a known type II topology. Each construct has an SV5 tag adjacent to the BAP tag, except for BAP-H2a, which has a BAP tag at the N-terminus and a myc tag at the C-terminus. *C*, S1R-BAP or BAP-S1R was expressed in HEK293 cells together with BirA or sec-BirA as indicated, and cells were incubated with biotin. Cell lysates were subjected to immunoblot with anti-V5, showing total expression or Strep-HRP to detect biotinylated species. On the *left*, the migration of MW markers in kDa. *D*, similar to (*B*) but with cells expressing H2a-BAP (*upper panels*) or BAP-H2a (*lower panels*). Additional controls show expression of only BirA or sec-BirA. *E*, relative biotinylation was calculated by dividing the Strep-HRP signal obtained for each sample by the total protein signal, the results attained for each protein with BirA + sec-BirA were then considered 100% for comparison purposes. The graph represents an average of four independent experiments for S1R constructs and three for H2a ±SD, *p* values ∗∗ =0.007, ∗∗∗ <0.0002, NS: nonsignificant >0.05. Student's *t* test (paired, two-tailed).

The crystal structure of the human sigma-1 receptor indicated the existence of only one TMD near the N-terminus of the protein (20). Previous reports had suggested one or two TMDs (4, 21, 22, 23). Furthermore, analyses of the S1R protein sequence (Fig. 1*A*) by different topology prediction methods estimate a surprisingly wide range of topologies, with one to three TMDs, and both possible orientations of the N-terminus, toward the ER lumen or toward the cytosol (Fig. S1).

If indeed the S1R has a single TMD, given the lack of a signal peptide, it could adopt either a type II orientation (C-terminus in the ER lumen) or a type III orientation (N-terminus in the ER lumen). Moreover, both the prediction published with the 3D structure (20) and the combined prediction of the CCTOP server (24) (Fig. S1) suggest a type III orientation. To further inspect the S1R orientation, we first used a system that allows *in vivo* labeling in cells and sensitive detection of protein epitopes in the ER lumen or in the cytosol. This system uses a specific biotinylation reaction by the *Escherichia coli*-derived biotin-ligase BirA (25) on a 15 aa long biotin acceptor peptide (BAP), which is linked to the protein of interest. BirA can be expressed in the cytosol in mammalian cells, reacting only with BAP exposed to the cytosol (26). In contrast, a luminally expressed BirA (sec-BirA) labels BAP when exposed to the ER lumen. We have made constructs expressing BAP-tagged S1R, with the tag on the N-terminus (BAP-S1R) or C-terminus (S1R-BAP). If the protein has a type II orientation, S1R-BAP should be biotinylated by sec-BirA and not by BirA and vice versa for BAP-S1R. Conversely, if it has a type III orientation, S1R-BAP should be biotinylated by BirA and not by sec-BirA and vice versa for BAP-S1R (Fig. 1*B*). An SV5 tag was added next to the BAP tag, to detect the protein independently of biotinylation. The tagged proteins were expressed in HEK293 cells together with BirA or sec-BirA, and the cells were incubated with biotin. Cell lysates (prepared in the presence of N-ethylmaleimide to prevent postlysis biotinylation) were immunoblotted with anti-V5 to detect total levels of the proteins, or with Streptavidin linked to HRP (Strep-HRP) to detect only biotinylated molecules. The results show biotinylation of S1R-BAP by sec-BirA and only to a minor extent (∼25% of total) by BirA, suggesting a mostly type II orientation (Fig. 1, *C* and *E*). This was similar to the results obtained with a BAP-tagged construct made with a protein known to have a type II orientation, asialoglycoprotein receptor H2a (27) (Fig. 1, *D* and *E*). The small amount of labeling by BirA probably reflects molecules that have undergone retrotranslocation toward ERAD ((26, 28) and our unpublished results). In contrast to S1R-BAP, BAP-S1R was biotinylated to a similar extent by both BirA and sec-BirA (Fig. 1, *C* and *E*). This result was surprising and might be due to the N-terminal tag affecting the membrane orientation of the protein, giving equal probability for insertion in the two opposite orientations. This effect of the tag might explain the conflicting results in other studies that used S1R with other tags (4, 21, 29, 30). The orientation of H2a with a BAP-tag in the N-terminus (BAP-H2a) was affected to some degree, but still showed a predominant type II orientation (63% of total BAP-H2a) (Fig. 1, *D* and *E*).

**Figure 2:**
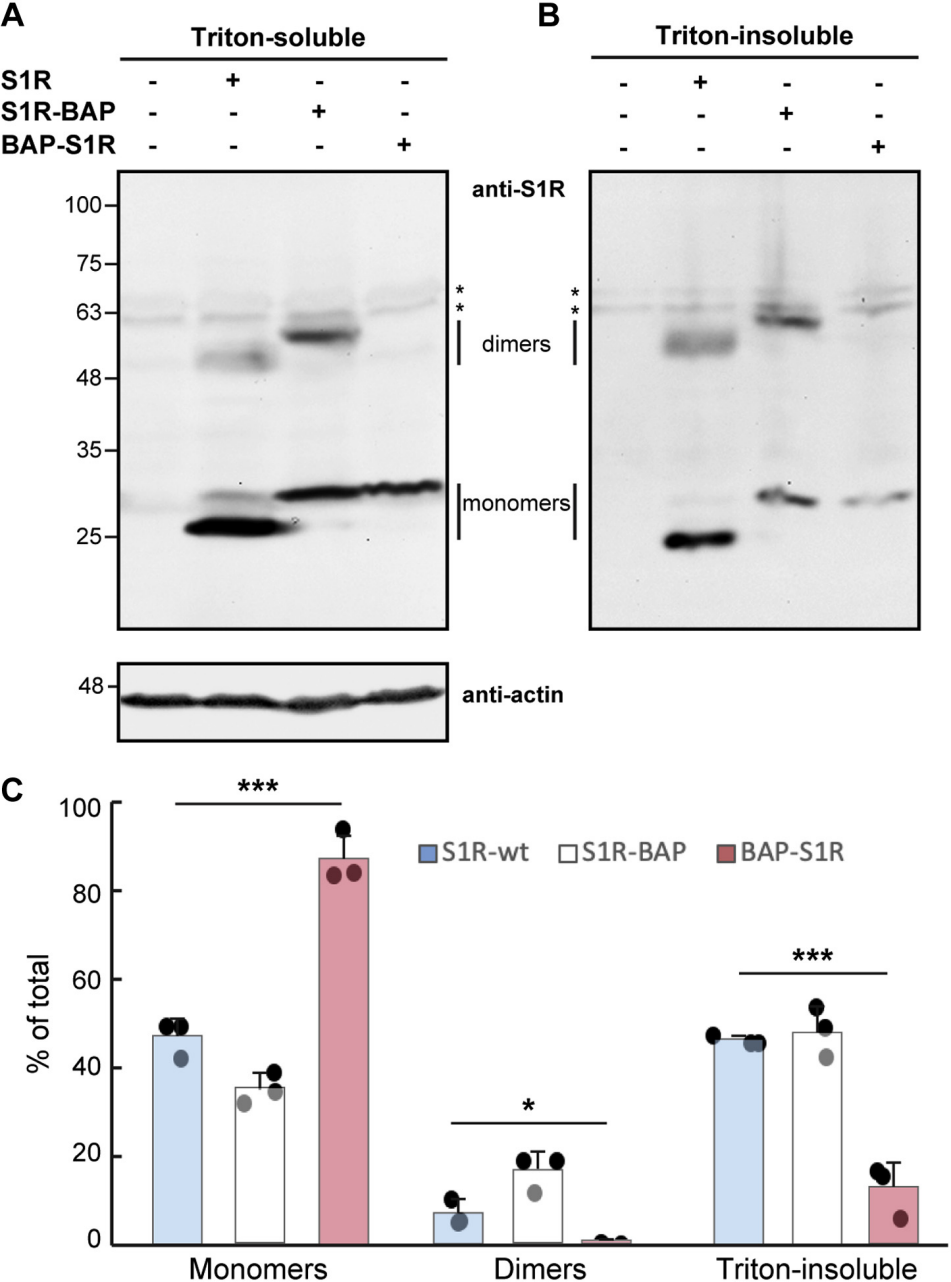
**N-terminal tagging affects S1R oligomerization.***A* and *B*, Triton-soluble (*A*) and Triton-insoluble (*B*) protein samples, from untransfected S1R−/− HEK293 cells or expressing WT-S1R, S1R-BAP, or BAP-S1R were incubated with sample buffer on ice and run on 10% SDS-PAGE. Immunoblots were detected using anti-S1R antibody. *C*, S1R monomers and dimers were quantified from (*A*) and high-order oligomers from the sum of all S1R signals in (*B*). The graph shows the % of total (sum of S1R signals in both (*A*) and (*B*)) for each species and is an average of three independent experiments ±SD. BAP-S1R showed significantly less dimers and high-order oligomers. *p* values ∗ =0.02, ∗∗∗ <0.0006. Student's *t* test (paired, two-tailed).

We analyzed whether the tags affect the oligomerization of the S1R. For this, we separated Triton-insoluble species (higher order oligomers (31)) and kept the lysates at 4 °C before SDS-PAGE, which prevents dissociation of S1R dimers (32). To avoid the formation of mixed oligomers, we performed these experiments by expressing the constructs in HEK293 cells where S1R was deleted using CRISPR/Cas9 technology (S1R−/−) (15). Whereas C-terminal BAP tagging caused a small increase in dimerization and did not affect association into higher-order oligomers, N-terminal tagging reduced significantly the presence of dimers (Fig. 2, *A* and *C*) and Triton-insoluble species (Fig. 2, *B* and *C*) compared with WT untagged S1R.

### A protease protection assay and the introduction of N-glycosylation sites in untagged S1R reveal a type II orientation

**Figure 3:**
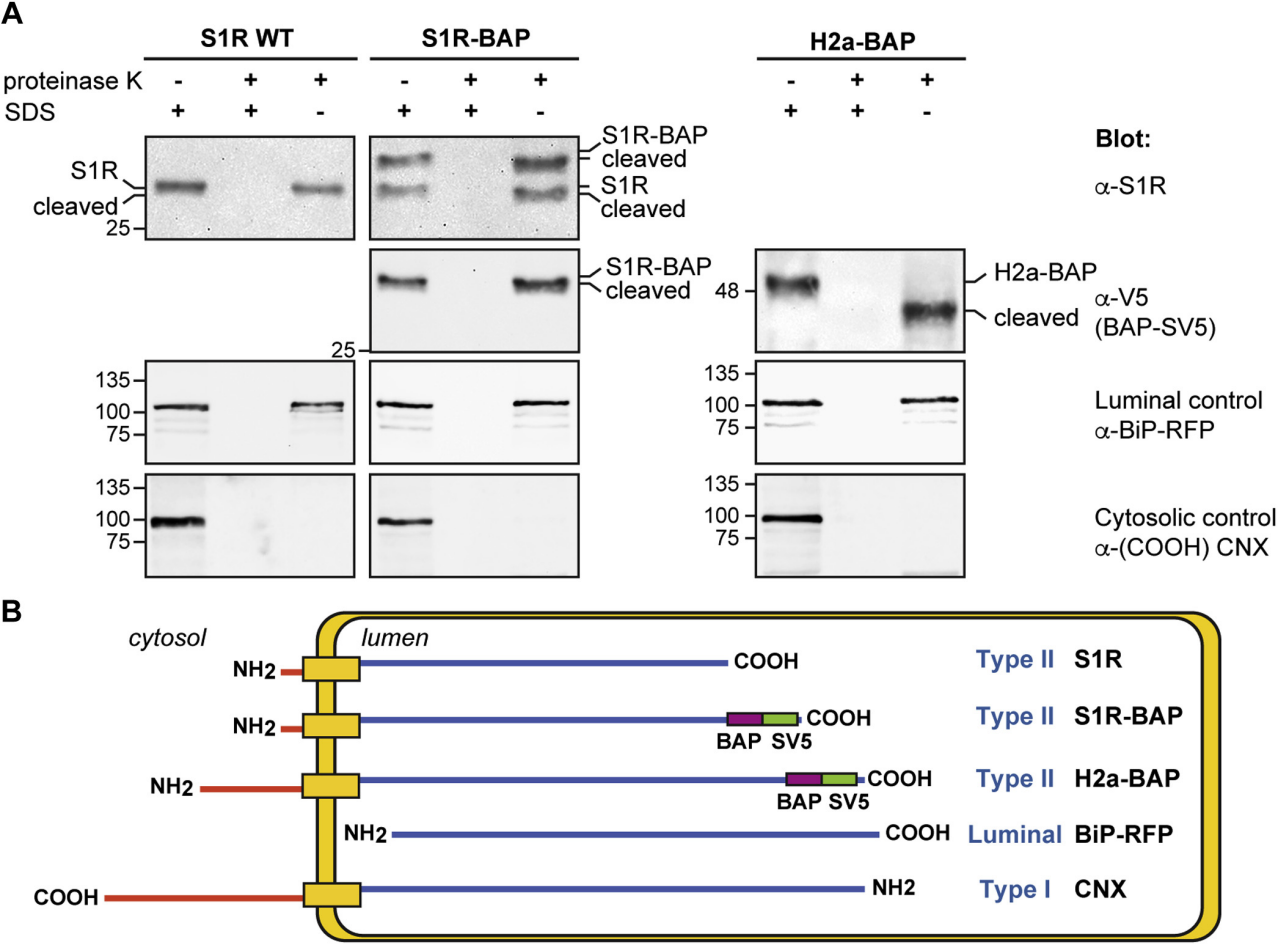
**Protease protection assay suggests a type II membrane topology.***A*, microsomes prepared by Dounce homogenization of HEK293 cells expressing either S1R-WT, S1R-BAP, or H2a-BAP were incubated as described in Experimental procedures, with or without proteinase K, before or after treatment with 1% SDS at 100 °C as indicated. Samples were then subjected to immunoblot with the antibodies indicated on the *right*. S1R and CNX were detected using antibodies recognizing epitopes toward the C-terminus. S1R-BAP and H2a-BAP were detected with antibodies against the SV5 tag at the C-terminus and the luminal control, BiP-RFP, was detected with anti-RFP. Microsomes treated with proteinase K without prior treatment with SDS protect the luminal and transmembrane regions of the proteins. For S1R, S1R-BAP, and H2a-BAP, a shift in migration due to cleavage by proteinase K of the cytosolic tails is indicated. Results are shown for a representative experiment of four independent repeat experiments. *B*, scheme of the results, which show type II membrane topology for S1R, S1R-BAP, and H2a-BAP (control for a type II membrane protein). The luminal control, BiP-RFP, was completely protected in the absence of SDS and CNX (control for a type I membrane protein) was digested in the absence or presence of SDS and no longer recognized by an anti-C terminal antibody.

Given the confounding effect of N-terminal tagging, we decided to determine the topology of untagged S1R. We applied a classical protease protection assay on microsomes prepared from S1R−/− HEK293 cells transfected with WT S1R and compared with S1R-BAP and H2a-BAP. The microsomes were treated with proteinase K after treatment or not with 1% SDS. All three proteins were completely digested in the presence of SDS and were protected from the protease in the absence of SDS (Fig. 3*A*). Both S1R and S1R-BAP showed a small shift, using anti-S1R or anti-V5 antibodies for the latter, indicating a cleavage by proteinase K of a peptide of ∼1.3 kD, which is consistent with digestion of the small cytosolic tail in a type II orientation (Fig. 3*B*). H2a-BAP showed a larger cleavage of ∼6 kD, which is the size of its cytosolic tail. As controls, a luminal protein, BiP-RFP showed complete protection in the absence of SDS and calnexin (CNX), a type I membrane protein, was no longer recognized by an anti-C terminal antibody, in the absence or presence of SDS (Fig. 3*A*).

**Figure 4:**
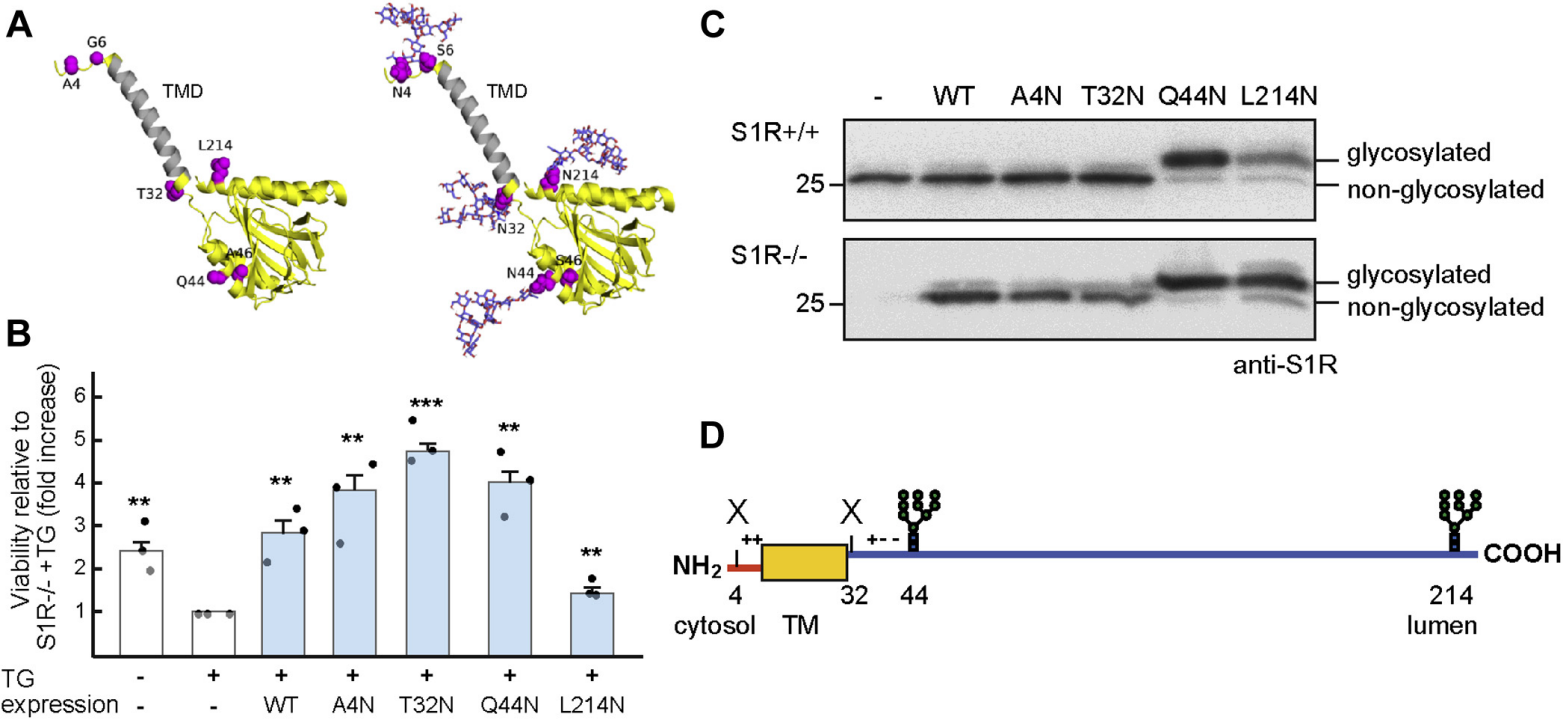
**An approach introducing N-glycosylation sites into untagged S1R indicates a type II membrane topology.***A*, 3D structure of the human S1R (*left*) and of the same structure showing potential N-glycans (Man_9_GlcNAc_2_) (shown as sticks) (*right*), at glycosylation sites introduced into four different constructs. The protein is shown as a *yellow ribbon*, with the positions targeted for mutations (4, 6, 32, 44, 46, and 214) shown as magenta spheres and labeled. The transmembrane domain α-helix (TMD) is colored in *gray*. The N-terminus of the protein is on the top. *B*, S1R−/− HEK293 cells, transfected with WT S1R or with the N-glycosylation site constructs, mutations in parenthesis: A4N (A4N and G6S), T32N (T32N), Q44N (Q44N and A46S), and L214N (L214N) or mock transfected were treated with 2 μg/ml TG for 24 h and cell viability was tested compared with the treated untransfected cells using a RealTime-Glo MT cell viability assay. The graph represents the mean of three independent experiments ±SEM, *p* values (compared with treated untransfected cells) ∗∗ <0.01, ∗∗∗ =0.00015. Student's *t* test (paired, two-tailed). *C*, S1R+/+ (*top*) or S1R−/− (*bottom*) HEK293 cells were transfected with WT S1R or with the N-glycosylation site constructs or left untransfected and subjected to immunoblotting with an anti-S1R antibody. *D*, scheme of the results of the experiment in (*C*). The N(44)LS and N(214)TT sites were glycosylated, while the other two introduced sites, N(4)VS and N(32)QS, were not, suggesting a type II membrane protein topology. Charges in amino acids in the juxtamembrane region are shown.

To discard any changes that might take place during the manipulation needed for the preparation of microsomes, we used another approach to reveal the topology of untagged S1R in intact cells. S1R is a nonglycosylated protein. To analyze the orientation of untagged S1R, we made different constructs where individual N-glycosylation sites were introduced and expressed them in S1R−/− HEK293 cells. As N-glycosylation takes place only in the ER lumen, these constructs should reveal the orientation of the protein. N-glycosylation does not affect the normal topology of the protein as the N-glycans are added only after translocation into the ER lumen of a given segment containing an N-glycosylation sequon. N-glycosylation sites were introduced at exposed hydrophilic regions and with minimal effects on polarity (Fig. 4*A*). A viability assay was used to test if the constructs expressed functional S1R, which would protect cells from ER stress-induced cytotoxicity caused by long-term incubation with thapsigargin (TG). S1R functionality would reflect a native conformation. All constructs protected the cells to a similar or slightly higher extent than WT S1R, except for S1R L214N, which showed a lower, although still significant protection (Fig. 4*B*). Constructs with N-glycosylation sites near the N-terminus, S1R A4N and S1R T32N, resulted in proteins with no change in migration in SDS-PAGE compared with WT S1R, suggesting no glycosylation at these sites (Fig. 4*C*). In contrast, a construct with a more C-terminal N-glycosylation site, S1R Q44N, and one near the C-terminus, S1R L214N, resulted in a slower migration in SDS-PAGE compared with WT S1R, consistent with N-glycosylation at these sites. We observed the same differences between these proteins when expressed in S1R+/+ or S1R1−/− cells (Fig. 4*C*). Only a minority of S1R Q44N (14%) and S1R L214N molecules (18%) were not glycosylated, measured in S1R1−/− cells. These results suggest that most of the protein, including its C-terminus, is in the ER lumen. The T32N site is in the lumen, but too close to the TMD (residues 10–31) to be glycosylated, as a stretch of a minimum of 12 residues C-terminal to a membrane span is needed for N-glycosylation to take place (33) (Fig. 4*D*). An opposite orientation, with the N-terminus in the ER lumen would have resulted in no glycosylation for any of the constructs, as the S1R A4N is also too close to the membrane span. The results suggest a type II transmembrane protein topology.

### Neither S1R agonists nor ER stress changes the topology or the ER exit of S1R constructs

**Figure 5:**
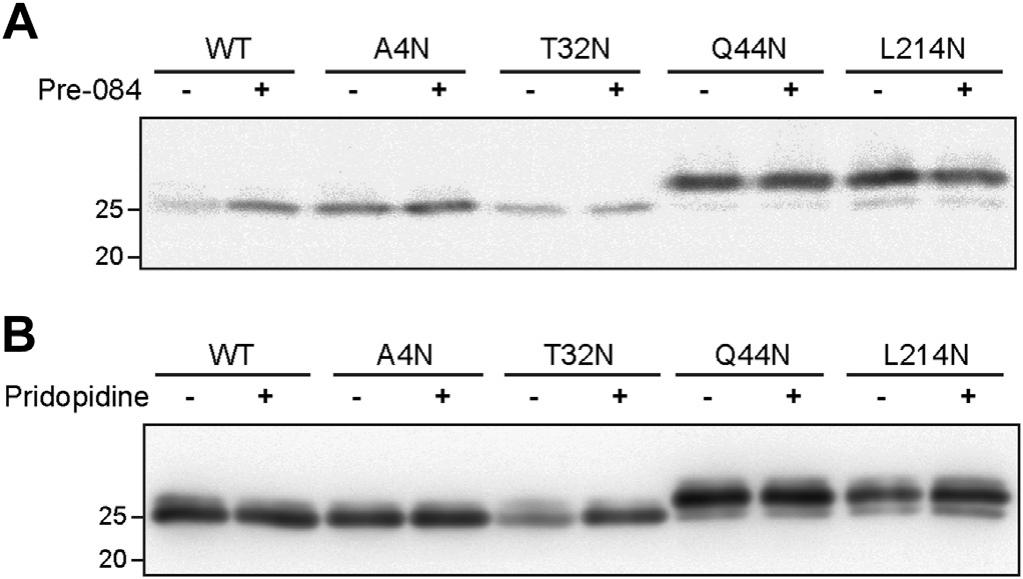
**S1R topology does not change in the presence or absence of S1R agonists.***A*, S1R−/− HEK293 cells expressing WT S1R or the N-glycosylation site constructs were treated with 100 nM Pre-084 for 16 h or left untreated. Cell lysates were immunoblotted with anti-S1R. No change is observed in the glycosylation patterns. *B*, similar to (*A*) but using 3 μM pridopidine instead of Pre-084.

To test whether the topology or orientation of the S1R constructs was affected by an S1R agonist, we incubated S1R−/− HEK293 cells expressing WT S1R or the constructs with added N-glycosylation sites with the S1R agonist Pre-084 (34). There was no change in the migration in SDS-PAGE for any of the constructs with or without Pre-084 incubation (Fig. 5*A*). Similar results were obtained with the S1R agonist pridopidine (35, 36) (Fig. 5*B*).

**Figure 6:**
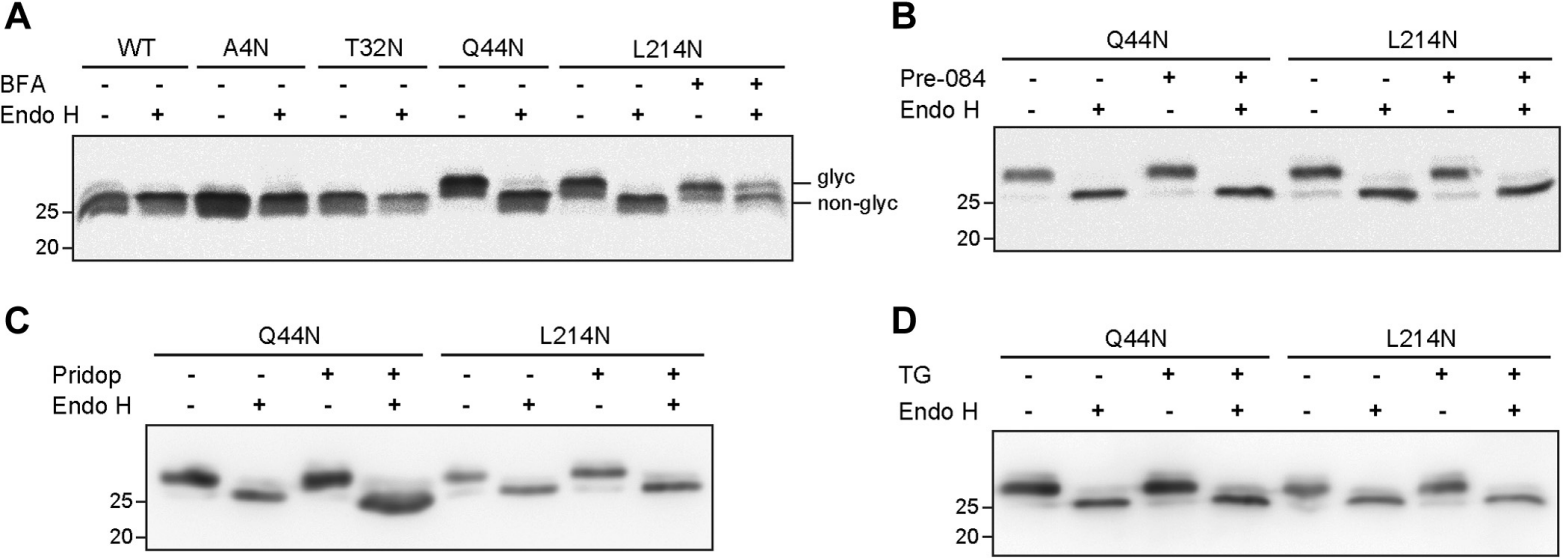
**S1R is ER-retained in the presence or absence of ER stress or of S1R agonists.***A*, lysates of S1R−/− HEK293 cells expressing WT S1R or the N-glycosylation site constructs were treated with or without endo H and immunoblotted with anti-S1R. The indicated samples were treated with 5 μg/ml BFA for 16 h before lysis, causing partial endo H resistance (two *right lanes*). *B*, similar to (*A*), but with cells treated with 100 nM Pre-084 for 16 h prior to lysis or left untreated. No change is caused by Pre-084 in the glycosylation patterns. *C*, similar to (*B*), but with cells treated with 3 μM pridopidine or left untreated. *D*, similar to (*B*), but with cells treated with 2 μg/ml TG for 16 h or left untreated.

We then analyzed the extent of ER exit of the proteins by measuring their resistance to endo-β-N-glycosaminidase H (endo H), which cleaves high-mannose type N-glycans. N-glycans are processed in the Golgi to complex-type sugar chains, acquiring resistance to endo H. Endo H treatment caused no change in the migration of WT, S1R A4N, and S1R T32N, as they are not glycosylated. In contrast, endo H treatment of S1R Q44N and S1R L214N changed their migration to a position similar to the WT S1R (Fig. 6*A*). There were few if any S1R Q44N and S1R L214N molecules that were resistant to endo H. To discard the possibility that even though they exit to the Golgi, these proteins carry high mannose N-glycans that are not converted to complex-type, cells were treated with brefeldin A (BFA). BFA causes ER-Golgi fusion and therefore delivers Golgi enzymes to the ER. When cells expressing S1R L214N were treated with BFA, the glycoprotein became partially (34 %) endo H resistant (Fig. 6*A*, rightmost lane). This result indicates that the S1R L214N N-glycan is capable of undergoing processing by Golgi enzymes to an endo H-resistant form, but is normally totally endo H-sensitive, suggesting complete ER retention.

As S1R agonists were reported to translocate the protein to or near the plasma membrane (17), we tested whether they cause any change in the endo H sensitivity. Neither Pre-084 nor pridopidine changed the endo H sensitivity of S1R Q44N or S1R L214N (Fig. 6, *B* and *C*). We also tested whether ER stress causes any changes, because the S1R is activated by ER stress (37). Incubation of cells with TG for 16 h did not change the endo H sensitivity of the glycosylated S1R constructs (Fig. 6*D*).

**Figure 7:**
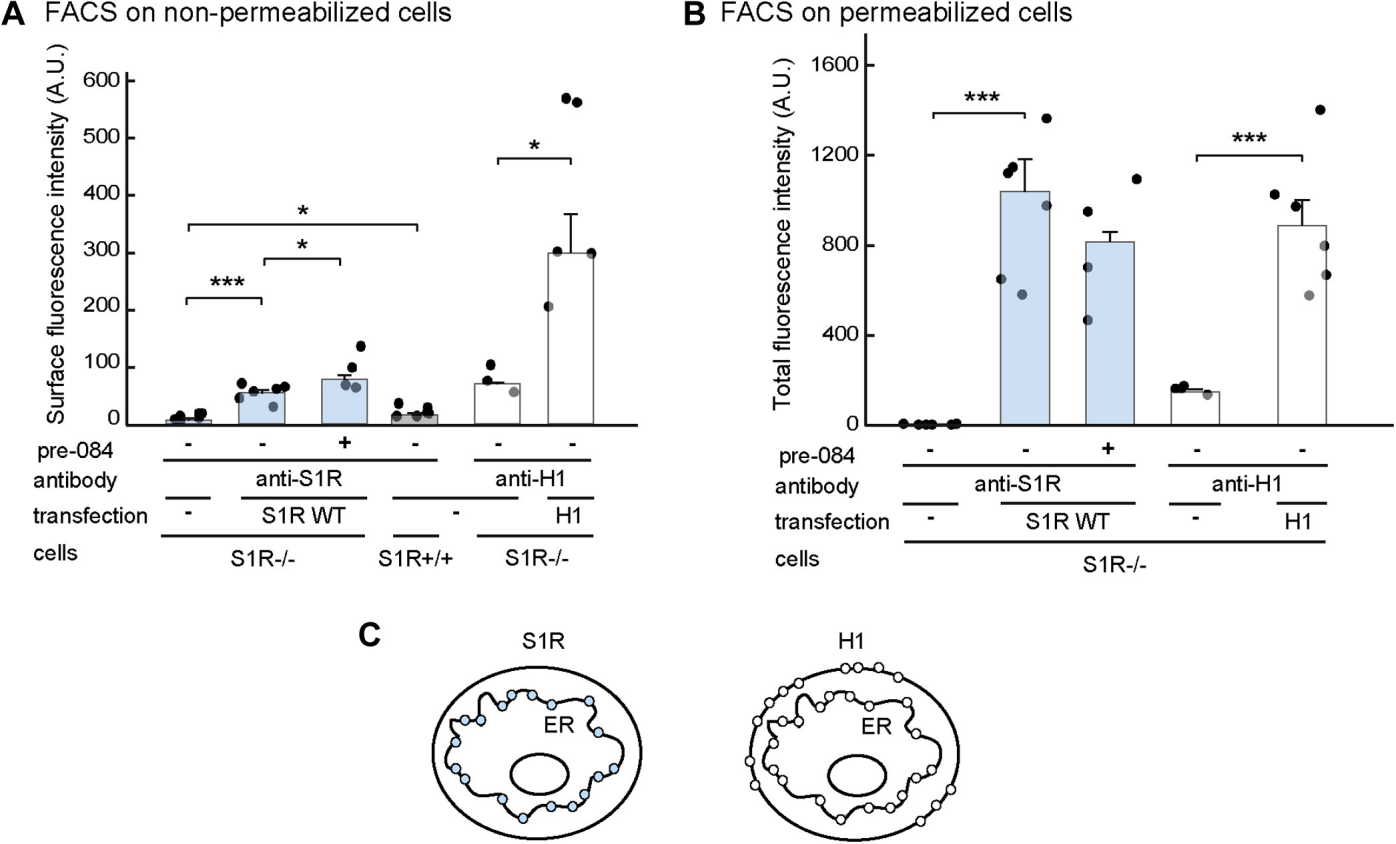
**FACS.***A*, cell surface S1R WT expressed in S1R−/− HEK293 cells or endogenous S1R in S1R+/+ HEK293 were analyzed in nonpermeabilized cells, incubated with anti-S1R at 4 °C followed by FACS, as described in Experimental procedures. Cells were pretreated or not with 100 nM Pre-084 for 16 h, as indicated. In parallel, as a positive control for cell surface expression, FACS was performed with S1R−/− HEK293 cells expressing asialoglycoprotein receptor H1, which is known to be partially exported to the cell surface. *B*, similar to (*A*) but performed on cells fixed and permeabilized with methanol. The graphs represent means of three independent experiments with samples in duplicates ± SEM, *p* values: Nonpermeabilized, compared with untransfected S1R−/− cells: S1R WT ∗∗∗=7E-5, endogenous S1R ∗ =0.05, H1 ∗ =0.02. S1R WT ± pre084 ∗=0.04. Permeabilized, S1R ∗∗∗ =1E-5, H1 ∗∗∗ =0.001. Student's *t* test (paired, two-tailed). *C*, scheme illustrating the similar expression of S1R and H1 on the ER membrane, whereas only H1 is expressed to a meaningful extent at the cell surface.

Although the results suggest that no S1R exits the ER, we wanted to test whether any molecules reach the cell surface. With this aim we first used fluorescence activated cell sorting (FACS) on nonpermeabilized S1R−/− cells expressing S1R or endogenous S1R in HEK293 cells. As a positive control, we measured the surface expression of an unassembled receptor subunit, asialoglycoprotein receptor H1, which can exit the ER and reach the cell surface but only to a modest extent (27). A very minor cell surface signal, although statistically significant, was observed for both transfected and endogenous S1R using anti-S1R antibodies (which recognize an epitope that should be exposed if S1R reaches the cell surface (Fig. 7*A* and Fig. S2)). In contrast, H1 showed strong surface fluorescence. When analyzing permeabilized cells, the signals obtained for S1R and H1 were of similar magnitude (Fig. 7*B*). The ratio of cell surface (nonpermeabilized cells) to total (permeabilized cells) was ∼30% for H1 and only ∼4% for S1R. Cell incubation with Pre-084 caused only a small increase in this ratio. We also analyzed the cell surface expression of BAP-S1R, reasoning that the altered topology caused by an N-terminal tag might affect the ER retention, but it also gave a very minor cell surface signal in the absence or presence of the agonist (Fig. S2). Therefore, only a minor fraction of S1R, if any, appears to reach the cell surface (Fig. 7*C*).

**Figure 8:**
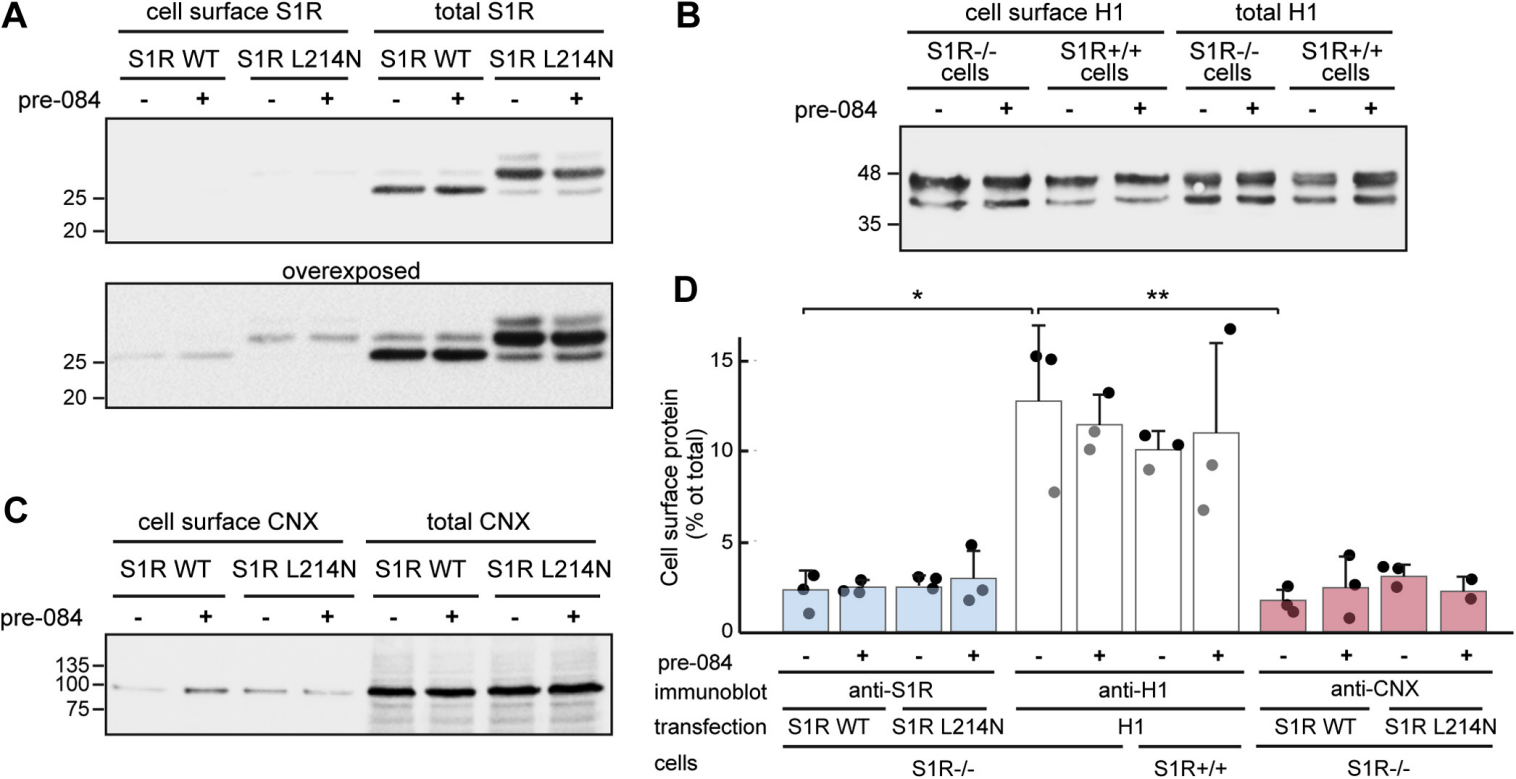
**Surface biotinylation shows that little if any S1R reaches the cell surface, similar to an ER chaperone.***A*, S1R−/− HEK293 cells expressing WT S1R or the L214N construct were treated with or without 100 nM Pre-084 for 16 h, followed by cell surface biotinylation with sulfo-NHS-LC-Biotin. Ten precentage of the cell lysates were immunoblotted with anti-S1R (total S1R). The rest of the lysates were precipitated using streptavidin-agarose beads before immunoblotting (cell surface S1R). The *lower panel* is an overexposure of the blot from the *upper panel*. *B*, similar to (*A*) but with S1R−/− or S1R+/+ HEK293 cells expressing H1. *C*, the immunoblot in (*A*) was reacted with anti-CNX antibodies, showing an expected very low percent of surface expression of the ER chaperone. *D*, quantitation of cell surface protein as a percent of total protein for S1R, H1, and CNX, from the experiments in *A–C*. The graph represents an average of three independent experiments ± SD, *p* values ∗ =0.015, ∗∗ =0.01. Student's *t* test (paired, two-tailed).

We then used another approach and applied a sensitive and specific surface biotinylation approach, using the membrane-impermeant reagent sulfosuccinimidyl-6-(biotinamido)hexanoate (sulfo-NHS-LC-Biotin). Biotinylated cell surface proteins from S1R−/− cells expressing WT S1R or S1R L214N were precipitated from cell lysates using streptavidin-agarose beads and immunoblotted with anti-S1R compared with total S1R. A weak signal of biotinylated S1R could be observed only upon overexposure of the blot and accounted for 2.2% of total WT S1R and 2.8% of total S1R L214N (Fig. 8, *A* and *D* and Fig. S3). There was no change when cells were treated with Pre-084. H1, when expressed in S1R−/− cells showed 12.7% of cell surface expression (Fig. 8, *B* and *D*). We also analyzed H1 expression in S1R+/+ cells, where it showed similar levels of cell surface biotinylated protein. To evaluate the significance of the low percent of surface S1R, we immunoblotted the same biotinylated samples with anti-CNX antibodies, as this is an ER-retained chaperone. Cell surface-biotinylated molecules of CNX were 1.7% of the total CNX in WT S1R expressing cells and 3.2% in S1R L214N expressing cells, not significantly different than the cell surface labeling obtained for the S1R (Fig. 8, *C* and *D*). Altogether, these results suggest that the S1R is an ER resident protein, not exiting the ER to any meaningful extent.

## Discussion

Previous studies of the S1R suggested a wide variety of topologies, including two TMDs, with both N- and C- termini facing the cytosol (21) or with both N- and C- termini facing the lumen (4). Studies of S1R truncation mutants suggest that the second hydrophobic segment, toward the C-terminus, may be peripherally attached, not being a TMD (22, 23). The recent determination of the crystal structure of the S1R indicates the existence of only one transmembrane span near the N-terminus. The probability that other hydrophobic segments would cross the membrane, as suggested by some of the bioinformatic predictions and by other studies, is extremely low.

Regarding the orientation of the S1R in the membrane, the studies also conflict. The crystal structure indicated one TMD; therefore the N- and C- termini of the protein must be on opposite sides of the membrane. However, the C-terminus was suggested to be in the cytosol in that study, from bioinformatic prediction (20). A recent study, based on BRET and ligand binding, confirmed that the N- and C- termini are on opposite sides, but was inconclusive as to the orientation (30). Our results with the BAP-tagged S1R, compared with those obtained with the untagged protein, suggest that at least N-terminal tagging can affect the orientation of the protein in the membrane. This is likely due to the short N-terminal tail, and thus the proximity of the tag to the TMD. N-terminal tagging was also reported to affect S1R oligomerization (32), which we confirmed with BAP-S1R. A study using an ascorbate peroxidase 2 (APEX2) tagging approach indicated a type II orientation, thus in this case the tagging did not seem to affect the orientation of the protein (29). Our study suggests that untagged S1R is in a type II orientation, with a short 9 amino acid N-terminal cytosolic tail, a TMD of 22 residues (10, 11, 12, 13, 14, 15, 16, 17, 18, 19, 20, 21, 22, 23, 24, 25, 26, 27, 28, 29, 30, 31), and a C-terminal portion, amino acids 32–223 in the lumen of the ER (Figs. 3 and 4). The classical protease protection assay indicates this clearly (Fig. 3).

The N-glycosylation mapping approach also yielded clear-cut results, with the site near the amino-terminus not being glycosylated, whereas the sites carboxy-terminal to the TMD are glycosylated, except for S1R T32N, which is too close to the membrane. This indicates that the S1R is a transmembrane protein with a signal anchor and a type II orientation, which is consistent with the positive-inside rule (38), as there are more positive residues in the amino-terminal, cytosolic side of the TMD (Fig. 4*D*). Only the TMHMM method gave this prediction (Fig. S1). The glycosylation results would also be compatible with Prodiv, but this would need 3 TMDs, which as mentioned above, is very improbable and is not in line with the crystal structure.

The minor cell surface biotinylation obtained for CNX (Fig. 8) could be due to a technical limitation of the experiment or to real cell surface expression of a small population of molecules of the ER chaperone, as has been reported previously in some cell types (39). Therefore, the similar minor biotinylation of the S1R suggests either no cell surface expression or only to a very minor extent (∼2%), comparable to that of an ER chaperone. Although this might be influenced by the fact that there is only one lysine in the S1R exposed domain that could be biotinylated, the FACS experiment confirms the finding. The results of the FACS experiment also yielded very low cell surface expression (∼4%). This suggests that the S1R cannot exit the ER to a significant extent by itself, although we cannot exclude that in some cell types it might exit the ER in complex with other protein(s).

The ER localization and type II orientation are consistent with S1R association with the ER luminal chaperone BiP and S1R activation upon dissociation from BiP (4). It is also in line with the main S1R function, modulation of the activity of the ER-localized inositol 1,4,5-triphosphate receptor (IP3R) (4, 40, 41).

S1R activation could hypothetically affect its topology, and it has been reported to translocate to the plasma membrane upon activation with agonists (17). However, our results indicate that neither the widely used S1R agonist Pre-084 (13, 34, 42) nor the high-affinity S1R ligand pridopidine (35, 36) affect the topology or ER exit of the S1R (Figure 5, Figure 6, Figure 7, Figure 8). Likewise, ER stress, which also activates the S1R (4), did not alter its ER retention (Fig. 6). It is possible that the report of plasma membrane location might have been due to technical shortcomings, and it might have been rather in internal membranes close to the plasma membrane, as mentioned above, likely at ER domains adjacent to the plasma membrane, as has also been described (19).

In conclusion, our results suggest that the S1R is an ER resident protein, not reaching the plasma membrane. The bulk of the S1R protein, spanning the C-terminal portion, is located in the ER lumen. This outcome concerning the S1R transmembrane orientation is the opposite of that predicted by some web-based algorithms and by the report on the crystal structure of the S1R (20). The fact that the S1R ligand-binding domain is thus inside the ER lumen predicts the properties that are shared by S1R ligands, such as hydrophobicity. Given the notable protective activity of the S1R in diseases, especially neurodegenerative diseases (12, 13, 14), our results could have important consequences in informing target-based therapies.

## Experimental procedures

### Materials

Streptavidin-agarose-beads, Brefeldin A (BFA), Biotin, sulfo-NHS-LC-Biotin, and thapsigargin were from Sigma. Pridopidine was a kind gift of Michal Geva at Prilenia Ltd. Pre-084 was from Tocris Bioscience. Endo H, HindIII, NotI, BamHI, and EcoRI restriction enzymes were from New England Biolabs (NEB).

### Plasmids and constructs

WT and the mutants of the S1R (A4N, T32N, Q44N and L214N) were produced by DNA synthesis and cloned (using EcoRI and BamHI restriction enzymes) into a pTwist EF1 alfa puro mammalian expression vector by Twist Biosciences. BAP-SV5 tag was added to the S1R plasmid, N-terminal tagged S1R (BAP-S1R) was cloned using the restriction enzymes BamHI and HindIII, C-terminal tagged S1R (S1R-BAP) was cloned using BamHI and NotI restriction enzymes. BirA and Sec-BirA plasmids were a kind gift from Gianlucca Petris and Oscar Burrone (ICGEB) (26). H2a-BAP was cloned in pcDNA3.1 using restriction enzymes EcoR1 and XbaI for H2a(G78R) followed by a C-terminal BAP-SV5 tag using restriction enzyme Xba1. BAP-H2a was cloned by introducing the BAP tag with restriction enzymes BamHI and EcoRI on the N-Terminus of H2a(G78R)-Myc-His in pcDNA3.1. H1 is cloned in pcDNA1 using HindIII and EcoRI restriction enzymes.

### Cell culture, media, and transfections

HEK293 S1R(−/−) cells (created using CRISPR/Cas9) and HEK293 cells were grown in DMEM supplemented with 10% bovine calf serum at 37 °C under 5% CO_2_. Transfections were carried out using the calcium phosphate method.

### BAP construct transfection and probing

BAP-tagged constructs of S1R and H2a were transfected, using the calcium phosphate method, in HEK293 cells with an equal amount of plasmids carrying either BirA (cytosolic biotin ligase) or Sec-BirA (ER luminal biotin ligase). Biotin (100 μM) was added 24 h post transfection for 1 h. The cells were then washed in PBS and lysed in Buffer A (1% Triton X-100, 0.5% NaDOC, and 1X protease inhibitors in PBS), containing 10 mM N-ethylmaleimide, to block BirA/Sec-BirA postlysis activity. The probing of biotinylated proteins on blots was performed using Strep-HRP in PBS-TWEEN (0.5%), followed with detection by enhanced chemiluminescence.

### Antibodies

Mouse monoclonal anti-S1R was from Santa Cruz Biotechnology. Mouse monoclonal anti-V5 was from GenScript, mouse anti-RFP, mouse anti-actin, and rabbit anti-calnexin were from Sigma. Strep-HRP, goat anti-mouse IgG-HRP, goat anti-rabbit IgG-HRP, goat anti-mouse Dylight 649, and goat anti-rabbit Dylight 488 were from Jackson-Immuno-Research Labs. Antibodies specific for peptides corresponding to the carboxyl termini of H1 were the ones used in a previous study (27).

### Treatments and immunoblotting

Pre-084 (100 nM for 16 h). Biotin (100 μM for 1 h). Pridopidine (3 μM for 16 h). Brefeldin A (BFA) (5 μg/ml for 16 h). TG (2 μg/ml for 16 h). Cell lysis was done in Buffer A. SDS-PAGE (12% except where indicated) and detection by enhanced chemiluminescence were done as previously described (43).

### Endo H treatment

Endo H treatment was carried out on the cell lysates according to New England Biolabs' (NEB) protocol. 10% of each lysate was denatured at 100 °C for 10 min, followed by incubation with Endo H for 1 h at 37 °C, in the appropriate buffers.

### Protease protection assay

HEK293 cells were transfected with BiP-RFP in combination with H2a-BAP, S1R-BAP, or S1R-WT plasmids. Transfected cells were collected 24 h post transfection and resuspended in Homogenization buffer (0.25 M Sucrose; 10 mM HEPES pH7.4) with protease inhibitors and subjected to homogenization using a Dounce homogenizer. Postnuclear supernatants (microsomes) from each sample were subjected to three different treatments: 1% SDS at 100 °C for 5 min, followed by incubation with or without 0.5 mg/ml proteinase K at 4 °C for 30 min. In the third treatment, microsomes were incubated with proteinase K at 4 °C for 30 min but omitting the prior boiling with SDS. The reactions were stopped with 12% TCA, TCA-precipitated pellets were boiled with sample buffer for 10 min at 100 °C and run on SDS-PAGE and subjected to immunoblotting.

### FACS analysis

HEK293 S1R (−/−) cells were transfected or not with S1R WT or H1 containing plasmids. Pre-084 (100 nM) was added to some samples 4 h posttransfection and incubated overnight. Twenty-four hours posttransfection, cells were collected, centrifuged, and resuspended in complete DMEM +0.1% NaN3 (to inhibit protein internalization). In total, 0.25–0.5 × 10^6^ cells were used per sample. After centrifugation, primary antibodies (mouse anti-S1R and rabbit anti-H1) were added directly to cell pellets for 45 min. After rinsing with DMEM +0.1% NaN3, secondary antibodies (1:50) were added to each tube for 45 min (Goat anti-mouse Dylight 649 or Goat anti-rabbit Dylight 488). Cells were rinsed and resuspended in PBS+0.1% NaN_3_ and kept in the dark until FACS analysis. The whole procedure was performed at 4 °C to prevent protein internalization.

A similar procedure was performed for permeabilized cells except that after collecting they were fixed and permeabilized with 100% methanol for 20 min at –20 °C.

### Cell surface biotinylation and streptavidin-precipitation

Pre-084 (1 μM) was added to the cells 4 h posttransfection and incubated overnight. Twenty-four hours posttransfection, cells were washed with PBS and incubated (rocking) with sulfo-NHS-LC-Biotin (0.5 mg/ml) for 45 min at 4 °C. Biotinylation was stopped using NH_4_Cl (50 mM) for 10 min at 4 °C, and the cells were washed with PBS. Cell lysis was performed in Buffer A for 30 min on ice. After centrifugation at 25,000*g* in 4 °C for 30 min, 10% of total lysate was saved, the rest of the lysate was incubated with streptavidin-agarose beads overnight. After washing three times with Buffer D (0.5% Triton X-100, 0.25% NaDOC, and 0.5% SDS in PBS), bound proteins were eluted with sample buffer at 100 °C for 5 min and immunoblotted.

### Cell viability assay

The cell viability assay was performed on transfected HEK293 S1R (−/−) cells, using the protocol for endpoint assay format of the RealTime-GloTM MT Cell Viability Assay (Promega). A final cell density, within the linear range, of 3000 cells per sample was chosen. Duplicates of samples seeded into opaque-walled assay plates 24 h posttransfection were treated with TG for 24 h. MT Cell Viability Substrate and NanoLuc Enzyme were diluted in the cell culture medium (2×) and added to the samples and incubated for 10 min at 37 °C. Luminescence from samples was measured using a plate-reading luminometer, Synergy H1 Hybrid Micro Reader (Biotek), with an integration time of 0.25–1 s per well.

### Determination of S1R oligomerization

S1R−/− HEK293 cells were transfected with either S1R-WT plasmid, N-terminal tagged S1R (BAP-S1R), or C-terminal tagged S1R (S1R-BAP) using the calcium phosphate method. The cells were collected 24 h posttransfection and lysed in Buffer A. After centrifugation at 25,000*g* in 4 °C for 30 min, the supernatants (Triton-soluble proteins) were transferred to a fresh tube leaving the pellet (Triton-insoluble proteins). Sample Buffer (0.05 M Tris-HCl pH 6.8, 0.1 M DTT, 2% SDS, 50% Glycerol, 0.1% bromophenol blue) was added to Triton-soluble and insoluble fractions and incubated on ice for 5 min. Samples were subjected to 10% SDS-PAGE immunoblotted and detected using anti-S1R antibody.

### Bioinformatic predictions and S1R structure model

The topology predictions were performed using the Constrained Consensus TOPology prediction server (CCTOP) (24). The structure of the human S1R monomer was taken from the Protein Data Bank (PDB ID 5hk2, chain A), and missing atoms were added using WHAT-IF (44). The residues at positions 4, 6, 32, 44, 46, and 214 were then mutated to Asn, and hydrogen atoms were added to the structure using MolProbity (45). A Man_9_GlcNAc_2_ glycan chain was added to positions 4, 32, 44, and 214, using the Glycan Reader module (46) of the CHARMM GUI web server (47). The glycan at N214 extends toward the TMD. However, a hinge motion around the region linking the TMD to the C-terminal region of the protein may allow rotation away from the membrane and its exposure to the ER lumen.

## Statistical analysis

The results are expressed as average ± SD or mean ± SEM as indicated. Student's *t* test (two-tailed) was used to compare the averages of two groups. Statistical significance was determined at *p* < 0.05 (∗), *p* < 0.01 (∗∗), *p* < 0.001 (∗∗∗).

## Data availability

This study includes no data deposited in external repositories.

## Supporting information

This article contains supporting information.

## Conflict of interest

The authors declare that they have no conflict of interest with the content of this article.
