# Supplementary material for: The Sigma-1 receptor is an ER-localized type II membrane protein
Source: J Biol Chem. 2021 Oct 11;297(5):101299. doi: 10.1016/j.jbc.2021.101299 (PMC8561001; doi:10.1016/j.jbc.2021.101299)
Supplement: Figures S1–S3 [file mmc1.pdf]

The Sigma-1 receptor is an ER-localized type II membrane protein

Neeraj Sharma, Chaitanya Patel, Marina Shenkman, Amit Kessel, Nir Ben-Tal and Gerardo Z. Lederkremer

Supplementary Material

Supplementary figures

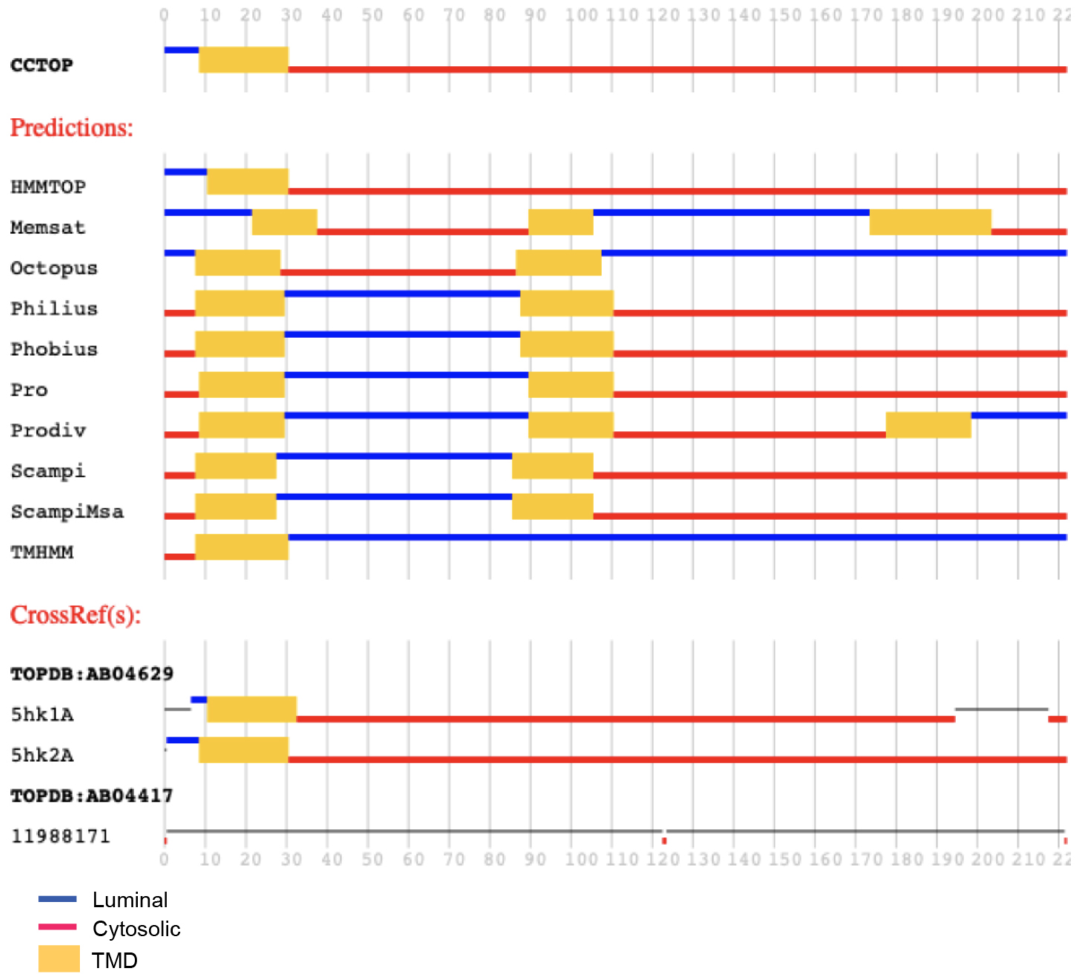

Suppl. Fig. 1. Bioinformatic predictions estimate conflicting topologies and number of transmembrane domains for the S1R. The CCTOP server predicts a type III membrane protein topology (top), based on a series of predictive methods yielding a variety of topologies.

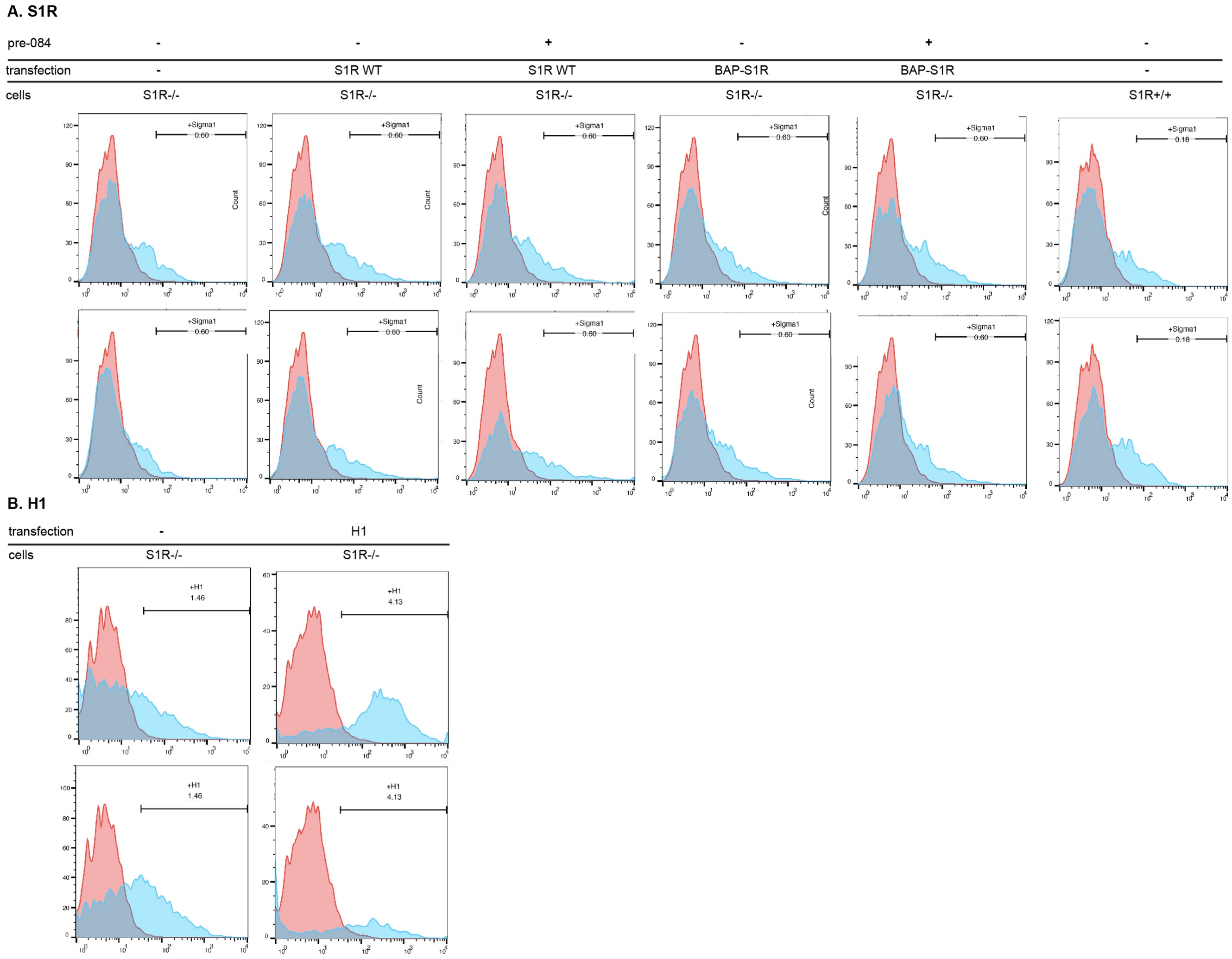

Suppl. Fig. 2. Raw FACS data of a representative experiment of 3 independent repeats corresponding to Fig. 7A. In addition, BAP-S1R was also analyzed showing a minor cell surface expression, similar to S1R WT.

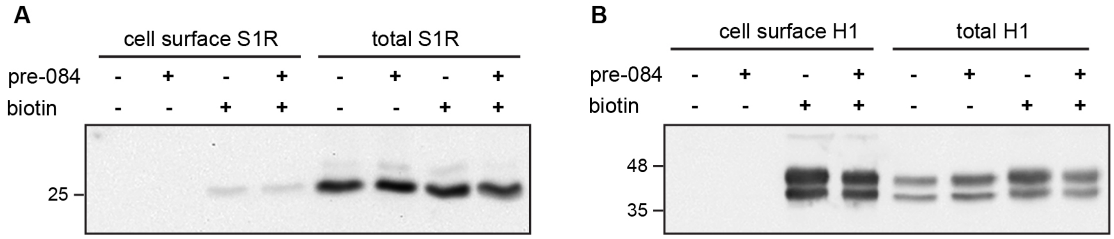

Suppl. Fig. 3. Experiment similar to that shown in Fig. 8, with S1R-/- HEK293 cells expressing WT S1R (A) or H1 (B). In this case controls were added where incubation with biotin was omitted, showing no detectable signal.
